# Insertion of a Specific Fungal 3′-phosphoadenosine-5′-phosphatase Motif into a Plant Homologue Improves Halotolerance and Drought Tolerance of Plants

**DOI:** 10.1371/journal.pone.0081872

**Published:** 2013-12-09

**Authors:** Meti Buh Gašparič, Metka Lenassi, Cene Gostinčar, Ana Rotter, Ana Plemenitaš, Nina Gunde-Cimerman, Kristina Gruden, Jana Žel

**Affiliations:** 1 Department of Biotechnology and Systems Biology, National Institute of Biology, Ljubljana, Slovenia; 2 Institute of Biochemistry, Faculty of Medicine, University of Ljubljana, Ljubljana, Slovenia; 3 Centre of Excellence for Integrated Approaches in Chemistry and Biology of Proteins (CIPKeBiP), Ljubljana, Slovenia; 4 Department of Biology, Biotechnical Faculty, University of Ljubljana, Ljubljana, Slovenia; Institute of Genetics and Developmental Biology, Chinese Academy of Sciences, China

## Abstract

Soil salinity and drought are among the most serious agricultural and environmental problems of today. Therefore, investigations of plant resistance to abiotic stress have received a lot of attention in recent years. In this study, we identified the complete coding sequence of a 3′-phosphoadenosine-5′-phosphatase protein, ApHal2, from the halotolerant yeast *Aureobasidium pullulans*. Expression of the *ApHAL2* gene in a *Saccharomyces cerevisiae hal2* mutant complemented the mutant auxotrophy for methionine, and rescued the growth of the *hal2* mutant in media with high NaCl concentrations. A 21-amino-acids-long region of the ApHal2 enzyme was inserted into the *Arabidopsis thaliana* homologue of Hal2, the SAL1 phosphatase. The inserted sequence included the META motif, which has previously been implicated in increased sodium tolerance of the Hal2 homologue from a related fungal species. Transgenic *Arabidopsis* plants overexpressing this modified *SAL1 (mSAL1)* showed improved halotolerance and drought tolerance. In a medium with an elevated salt concentration, *mSAL1*-expressing plants were twice as likely to have roots in a higher length category in comparison with the wild-type *Arabidopsis* and with plants overexpressing the native *SAL1*, and had 5% to 10% larger leaf surface area under moderate and severe salt stress, respectively. Similarly, after moderate drought exposure, the *mSAL1*-expressing plants showed 14% increased dry weight after revitalisation, with no increase in dry weight of the wild-type plants. With severe drought, plants overexpressing native *SAL1* had the worst rehydration success, consistent with the recently proposed role of *SAL1* in severe drought. This was not observed for plants expressing *mSAL1*. Therefore, the presence of this fungal META motif sequence is beneficial under conditions of increased salinity and moderate drought, and shows no drawbacks for plant survival under severe drought. This demonstrates that adaptations of extremotolerant fungi should be considered as a valuable resource for improving stress-tolerance in plant breeding in the future.

## Introduction

Water scarcity results in major agricultural losses worldwide, and is responsible for severe food shortages in developing countries [1]. Additionally, because of poor irrigation practices, water scarcity frequently leads to increased soil salinity, which can make large areas of arable lands useless for crop cultivation. High concentrations of salt affect all of the major processes in plants, from growth and photosynthesis, to energy production and lipid metabolism [2].

Despite significant efforts and some encouraging results, attempts to develop genetically modified salt-tolerant crops lines/cultivars have not yielded the desired results to date [3]. Manipulation of a wide variety of genes has been used, including genes encoding ion-transport proteins, genes involved in the synthesis of compatible organic solutes, antioxidants, and heat-shock and late embryogenesis abundant proteins, and genes encoding transcription factors for gene regulation [3], [4]. With the exception of ion transporters, the same groups of genes have been targeted in efforts to improve drought tolerance in plants [5]. The majority of used genes originated from either relatively stress-sensitive organisms or from prokaryotic organisms that are phylogenetically and functionally less related to plants. Stress-tolerant fungi do not have these disadvantages, however they have so far been largely overlooked as sources of stress-tolerance-conferring genes [6]. Here, we show that these fungal species can offer novel ways for the improvement of stress tolerance in plants.

The search for genes from *Arabidopsis thaliana* that can confer salt tolerance in yeast with impaired systems for efflux of sodium and lithium ions identified a gene known as *SAL1* (also *FRY1*) [7]. SAL1 is a bifunctional enzyme that hydrolyses 3′(2′)5′-bisphosphate nucleotides, such as 3′-phosphoadenosine 5′-phosphate (PAP) [8], [9], as well as the polyphosphoinositols, such as the inositol trisphosphates [10], [11]. SAL1 might also be responsible for repressing stress-responsive regulation via inositol 1,4,5-trisphosphate [10]–[12], although the 20-fold elevated PAP levels shown in mutants lacking SAL1 suggest that PAP is the main *in-vivo* substrate of SAL1 [13]. The nucleotidase activity of SAL1 might be responsible for modulation of the suppression of RNA silencing [8]. In addition, phenotype studies of *sal1* mutants have shown that the absence of the SAL1 protein results in reduced lateral root development [14], irregular leaf morphology [15], and light sensitivity [9], [16]. The *Arabidopsis* genome encodes four additional homologues of SAL1, as SAL2, SAL3, SAL4 and AtAHL [7], [17], [18], among which SAL1 is the major enzyme that hydrolyses PAP *in vivo* [19].

The homologue of SAL1 in *Saccharomyces cerevisiae* (Hal2) is an important determinant of halotolerance in this yeast [20]. Hal2 is encoded by the gene *HAL2*/*MET22*, and it is a sodium- and lithium-sensitive 3′-phosphoadenosine-5′-(PAP) phosphatase, which belongs to the phosphomonoesterase enzyme family (EC 3.1.3). Under normal conditions, Hal2 hydrolyses PAP, which is a by-product of sulphotransferation reactions and sulphur assimilation, and produces adenosine-5′-phosphate and inorganic phosphate [21], [22]. During salt stress, the enzyme activity of Hal2 is inhibited by sodium ions (IC_50_ = 20 mM), and PAP accumulates in the cell [21]. Consequently, this inhibits the sulphotransferases [23], RNA processing enzymes [24], and the sulphur assimilation pathway [21], which altogether leads to an inhibition of growth. Hal2 is even more sensitive to the lithium ions (IC_50_ = 0.1 mM) [21]. It has been shown that overexpression of *HAL2* improves salt tolerance of *S. cerevisiae* [20] and plants [25]. Recently, biochemical and genetic characterisation of two PAP phosphatases from the extremely halotolerant fungus *Hortaea werneckii* (*HwHAL2A*, *HwHAL2B*) identified a ‘META’ sequence motif, which is responsible for the unusually high sodium tolerance of both of these *H. werneckii* enzymes. This was supported by the observation that insertion of this META sequence into *HAL2* from *S. cerevisiae* enabled the yeast to grow at much higher environmental sodium concentrations (1.4 M) compared to overexpression of unmodified *HAL2* [26]. Homologues of *HAL2* have also been identified in the halotolerant *Debaryomyces hansenii* (*DHAL2*) [27] and in fission yeast (*TOL1*) [28].

The present study investigated the role of the newly identified PAP phosphatase from the polyextremotolerant black yeast *Aureobasidium pullulans*, ApHal2, in methionine biosynthesis and in tolerance to sodium. Furthermore, the META sequence from ApHal2, which is believed to be responsible for the tolerance of PAP phosphatase to sodium, was inserted into the *A. thaliana* homologue SAL1. The observed increase in salt and drought tolerance of the plants expressing the modified SAL1 phosphatase exemplifies the biotechnological potential of such fungi from extreme environments as gene donors for the improvement of stress tolerance of plants and possibly industrial microorganisms.

## Materials and Methods

### Strains and growth conditions

*Aureobasidium pullulans* (EXF-150) was isolated from the hypersaline waters of the Sečovlje solar salterns (Slovenia), and is preserved in the Ex culture collection of the Department of Biology, Biotechnical Faculty, University of Ljubljana (Infrastructural Centre Mycosmo, MRIC UL). These cells were grown at 28°C in a rotary shaker (180 rpm) in the defined YNB medium of 0.17% (w/v) yeast nitrogen base (YNB), 0.08% (w/v) complete supplement mixture (both Qbiogene), 0.5% (w/v) ammonium sulphate, 2.0% (w/v) glucose in deionised water, with NaCl added to different concentrations (0%, 2.5%, 5%, 7.5%, 10%, 13%; w/v), and the pH adjusted to 7.0. The cells were harvested in the mid-exponential growth phase using a 10-min centrifugation at 4000× *g*. For hypersaline stress, these *A. pullulans* cells were grown in YNB medium without added NaCl, to an optical density at 600 nm (OD_600_) of 1.0, harvested, and then resuspended in YNB medium with 10% NaCl (w/v). For hyposaline stress, the opposite order was used (growth at 10% NaCl; shock in medium without NaCl). Aliquots of the cell suspension were removed before and at the indicated times after the shock. The cells were separated from the growth medium by rapid filtration through a 0.45-µm-pore filter, and immediately frozen in liquid nitrogen.

The wild-type *S. cerevisiae* BY4742 (MATα; his3Δ1; leu2Δ0; lys2Δ; ura3Δ0) and the *S. cerevisiae HAL2*/*MET22* deletion strain (BY4742; YOL064c::kanMX4) were obtained from the Euroscarf Yeast Deletion Strain Collection, Frankfurt, Germany. Yeast cells were grown in YNB medium at 30°C, with constant shaking at 180 rpm.

### DNA and RNA isolation

Highly purified fungal genomic DNA was isolated from mid-exponential-phase cells grown in YNB medium according to a previously described cetyltrimethylammonium bromide chloroform/isoamyl alcohol extraction method [29]. For the total RNA isolation, *A. pullulans* cells were homogenised in liquid nitrogen with a pestle and mortar, followed by RNA isolation using the TRI Reagent (Sigma-Aldrich), according to the manufacturer instructions. RNA was extracted from mid-exponential-phase cells grown in YNB medium with different NaCl concentrations (as described above), and from cells exposed to hypersaline and hyposaline shock. Possible contaminating DNA was degraded with DNAse I (Fermentas), and the integrity and purity of the RNA was evaluated spectrophotometrically and by capillary electrophoresis (Agilent 2100 Bioanalyser). The cDNA from total *A. pullulans* RNA was synthesised using RevertAid H Minus First-Strand cDNA Synthesis kits (Fermentas).

Plant tissue (100 mg) was homogenised using a Tissuelyser (QIAGEN, Germany) and RNA was isolated with innuPREP Plant RNA kit (Analytic Jena, Germany). All of the samples were treated with DNAse (Invitrogen, USA) and analysed by gel electrophoresis and NanoDrop spectrophotometry (NanoDrop Technologies, USA), to determine the concentrations and purities of the isolated RNA.

### Cloning and sequencing of the *ApHAL2* gene from *A. pullulans*

A partial sequence of the *ApHAL2* gene had already been identified in a previous study [26]. The complete sequence of the gene was obtained using GenomeWalker Universal kits (Clontech), according to the manufacturer instructions, using oligonucleotide primers specific for adapters supplied in the kit and primers specific for known fragments of the genes. The primer sequences are listed in Table S1: gwaph2U (upstream), gwaph2Un (upstream nested), gwaph2D (downstream), and gwaph2Dn (downstream nested). The amplified products were sequenced by Macrogen Inc. (Korea), and assembled into a complete coding sequence. The nucleotide sequence has been deposited in GenBank under the accession number KC242234.

### Transformation of *A. thaliana* with *SAL1* and modified *SAL1*

The *SAL1* gene was amplified from *A. thaliana*, ecotype Col-0, cDNA (*Arabidopsis* Biological Resource Center, USA). PCR was performed with Velocity DNA polymerase (Bioline, USA) using the sal1_F and sal1_R primers (Table S1), which were designed on the U40433.1 sequence (Genebank). The amplified fragment was cloned into the pENTR D-TOPO plasmid, using the pENTR Directional TOPO Cloning kit and *Escherichia coli* TOP10 (both Invitrogen, USA). The sequence of the resulting pENTR::Sal1 plasmid was verified by Macrogene Inc. (Korea). Nucleotide sequence coding for mSAL1 (Table S2) was adapted for codon use in *A. thaliana* (Cherry, 1992) and ordered in pDONR221 (Invitrogen, USA) at GenScript (USA). Using Gateway LR Clonase II Enzyme Mix (Invitrogen, USA), the *SAL1* and *mSAL1* genes were cloned into the destination plasmid, pMDC32 [30] for gene overexpression in the plants, driven by the *35S* promotor. The resulting vectors pMDC32::Sal1 and pMDC32::mSal1 were selected in OmniMAX T1 Phage-Resistant *E. coli* (Invitrogen, USA) and transformed into ElectroMAX *Agrobacterium tumefaciens* LBA4404 (Invitrogen, USA). *A. thaliana* plants of ecotype Col-0 were transformed by the floral dip method [31], and surface-sterilised seeds of the transformants were selected on Murashige-Skoog medium supplemented with hygromycin. The plants were self-pollinated and the next generation seeds (T2) were collected to grow the plants for the analysis of the transgene expression levels and for the physiological studies.

### Functional expression of *Hal2* homologues in *S. cerevisiae*, and salt-tolerance assays

For the expression of the *HAL2* homologues in *S. cerevisiae*, the corresponding open-reading frames were amplified from *A. pullulans* gDNA or the pENTR::Sal1 and pDONR221::mSal1 plasmids, using the following primer sets (Table S1): for ApHAL2, primers aphal2_F and aphal2_R, containing EcoRI and BamHI restriction sites, respectively; for SAL1 and mSAL1, primers sal1_sc_F and sal1_sc_R, containing SmaI and KpnI restriction sites, respectively. For the control expression, *HAL2* was also amplified from *S. cerevisiae* gDNA using the primers hal2_F and hal2_R, containing SmaI and KpnI restriction sites, respectively. The resulting products were cloned into EcoRI/BamHI or SmaI/KpnI sites of the low-copy-number plasmid pRD53 (CEN, ARS, URA3, GAL1/10 promoter, Amp^R^), providing the plasmids pRD53::ApHAL2, pRD53::SAL1, pRD53::mSAL1 and pRD53::HAL2. The cloned *ApHAL2* and *HAL2* sequences were verified by sequencing (Macrogen Inc., Korea). Codon usage of the *A. thaliana* homologue gene *SAL1* and its modified version *mSAL1* was not optimised for *S. cerevisiae*.

Yeast cells were grown in yeast extract, peptone, dextrose (YPD) medium (1% yeast extract, 2% peptone, 2% glucose; pH 7.0) at 30°C and 180 rpm, to mid-exponential growth phase. They were then transformed with 1 µg of the pRD53_EV_, pRD53::ApHAL2, pRD53::HAL2 or pRD53::HwHAL2A [26] plasmids, using Alkali-Cation Yeast Transformation kits (Qbiogene). The transformants were selected on YNB plates without uracil (YNB-Ura) (Formedium). Positive colonies were grown in YNB-Ura medium supplemented with galactose (Gal), to mid-exponential phase, and adjusted to an OD_600_ of 0.5 for the functional complementation and stress-tolerance assays. Ten-fold serial dilutions (10^0^–10^4^) of the transformants in the corresponding medium were prepared and spotted in 3 µl aliquots onto YNB-Ura or YNB plates without uracil and methionine (YNB-Ura-Met), supplemented with Gal and different NaCl concentrations. The plates were incubated at 30°C for 3 to 5 days, and then scanned.

### Gene expression analysis

For the analysis of *ApHAL2* expression, real-time PCR with SYBR-Green-based detection was used. Approximately 100 ng cDNA was used as the template. The aphal2 primers did not form observable dimers. The reaction mix was prepared using the Power SYBR Green PCR Master Mix (Applied Biosystems), according to the manufacturer instructions. The thermal profile of the reaction was the following: 2 min at 50°C, 10 min at 95°C, 40 cycles of 15 s at 95°C and 1 min at 60°C, followed by a dissociation curve. Each reaction was performed in two biological and technical replicates. Expression values of the *ApHAL2* gene were standardised to the amount of the 28S rRNA gene fragment.

The levels of the transgene expression in the plants were determined with reverse-transcription qPCR (RT-qPCR) assays, quantifying mRNA for *SAL1* or *mSAL1* with custom designed primers and probes (Table S1), designed using the PrimerExpress 2.0 software (Applied Biosystems, USA). As a reference gene, *cox* was used (cytochrome c oxidase; *Arabidopsis* Information Resource [TAIR] accession number: ATMG01360.1), with a method adapted from one used for potato (*Solanum tuberosum*) [32], with the primers adapted for *A. thaliana* (Table S1). Another reference gene, human 18S rRNA (Applied Biosystems, USA) was introduced to monitor the RNA expression in the physiological experiments. Analyses were performed on two dilutions per sample, each in two replicates, using AgPath-ID One-Step RT-PCR kits (Ambion, USA). The reactions were carried out using a 50-µl reaction volume, with 30 min at 48°C for reverse transcription, followed by universal PCR conditions on an ABI 7900HT (Applied Biosystems, USA). The data were analysed with the SDS2.2.2 software (Applied Biosystems, USA), and the relative gene expression was determined as previously described [33]. The expression in individual samples was normalised to the expression of two endogenous control genes, with the results expressed as log_2_ of the ratio between the gene expression in the two samples. The relative amounts of RNA were calculated using the ΔΔCq method [34].

### Arabidopsis stress tolerance assays

Two sets of physiological studies were carried out. First, the effects of salt were determined for the three genotypes (the wild-type, sal1 and msal1 lines) in tissue culture. The effects of salt were measured in terms of the root length and the leaf surface area. In the second part, the effects of short and long exposures to drought conditions were determined on soil-grown plants.

(i) To test for differences in the halotolerance between genotypes, the plants were grown on vertical plates containing MS medium with 3% sucrose and 1% agar, supplemented with 0, 50 and 100 mM NaCl. The wild-type and 10 *sal1* lines and 11 *msal1* lines were tested, each with 20 surface-sterilised seeds per growth condition. After 48 h at +4°C in the dark, the plants were cultivated in a growth chamber with an 8-h day (70-90 µmol·m^−2^·s^−1^ at 19°C)/16-h night (17°C) cycle. The root lengths of three-week-old plants were measured, and the plants were photographed under the same light conditions. The area of the green shoots was estimated for each plate, with pixel counting using the Adobe Photoshop programme (Adobe, USA). In an additional experiment, the plants were grown in soil, and after three weeks they were watered every second day with 1/8 MS containing 0, 50, 100, 150 and 200 mM NaCl. The wild-type and two *sal1* lines and three *msal1* lines were tested.

(ii) Under drought, the experimental plants were cultivated in a growth chamber with an 8-h day (120–150 µmol·m^−2^·s^−1^ at 20°C)/16-h night (18°C) cycle at 75% relative humidity. Four-week-old plants were transferred to 150-mL pots containing potting soil, and they were equally watered with 25 mL water. They were rewatered with the same amount of water 2 days later, and transferred to a green house. Sixty plants were grown for each of the tested lines (wild-type, 3 *sal1*, and 3 *msal1* lines): 20 were watered normally, while 20 were not watered for two weeks (moderate drought), and 20 not for three weeks (severe drought). Following the drought stress, the plants were watered normally for a week [35], after which their green biomass and dry weight were measured (after drying at 105°C for 24 h).

### Bioinformatics analysis

Sequence alignments were performed using ClustalW (http://www.ebi.ac.uk/clustalw/) [36], with the default settings.

The three-dimensional models of ApHAL2 and SAL1 were built by homology-based protein-structure modelling, using the programme Swiss Model [37]. The input were the crystallographic template structure of *S. cerevisiae* HAL2 (Protein Data Bank (PDB), 1QGX), and the ApHAL2 (DQ519094.1) and SAL1 (ID 836519) sequences, aligned with Align X (Vector NTI 10, Invitrogen, USA). The models were examined in PredictProtein PHD (www.expasy.org) and visualised with UCSF Chimera [38].

### Statistical analysis

To determine the effects of NaCl on root growth in the three genotypes tested (wild-type, *sal1* and *msal1* lines), we first defined root length into four categories: 0 (0.0–0.9 cm), 1 (1.0–1.9 cm), 2 (2.0–2.9 cm) and 3 (3.0–3.9 cm). The maximal root length was limited by the size of the petri dishes, and thus could not exceed 4 cm. Logistic regression was used to measure the influence of varying the NaCl concentrations, as well as the different genotypes, on root length. To simultaneously monitor the effects of NaCl on root length for a specific genotype, the interaction term was used in the model.

To determine the differences in leaf surface area for the genotypes tested (wild-type, *sal1* and *msal1* lines) when exposed to the various salt concentrations (0, 50 and 100 mM NaCl) we used mixed-effects modelling. The variability within the 21 different lines included in the experiments was used as the source of random variation. The salt concentration was defined as an ordered factor. The interaction term between salt and genotype was not used in the final model, as this was not significant.

To test for effects of mild or severe drought exposure on the soil-grown plants, mixed-effects modelling was again used, with the transgenic line used as the source of the random variation. The data for the mild and severe drought stress were analysed separately. For both of these stress conditions, the dry weight and the fresh weight/dry weight ratio were modelled as a function of the drought condition (normal plant watering, mild/severe drought exposure) and genotype tested (wild-type, *sal1* and *msal1* lines). The water potential was measured in the plants exposed to severe drought conditions, and tested, again using mixed-effects modelling with the line used as the source of random variation.

All of the statistical data analyses were performed in R [39] using the libraries necessary for the logistic regression [40] and mixed-effects modelling [41].

## Results

### *ApHAL2*, the *HAL2* homologue from *A. pullulans*

**Figure 1:**
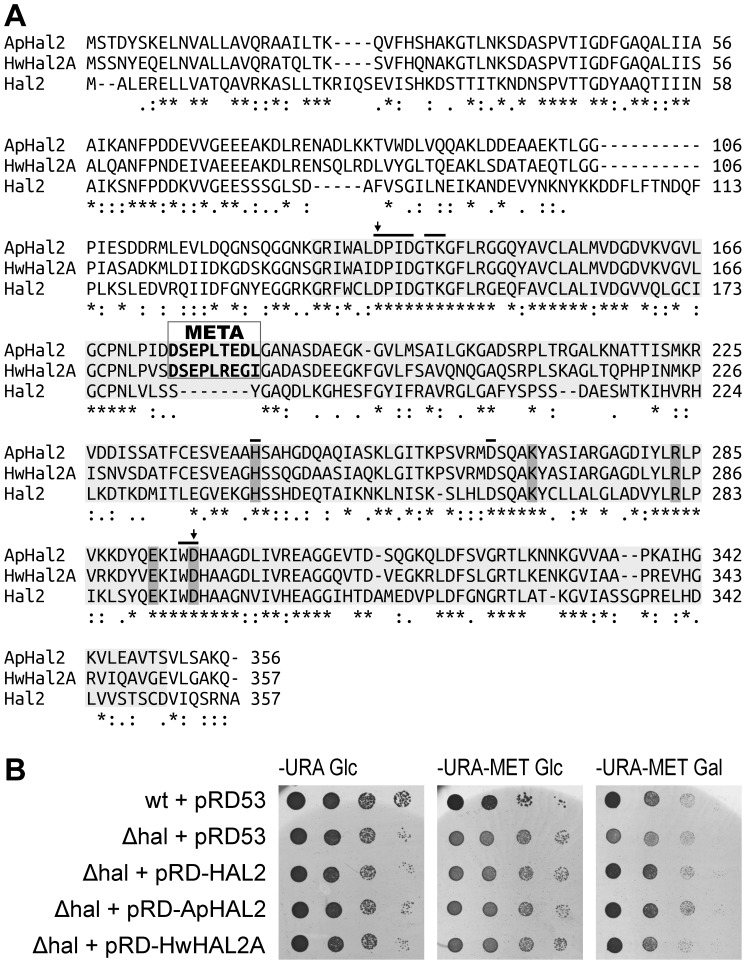
The *A. pullulans* 3′-phosphoadenosine-5′-phosphatase, ApHal2. (A) Amino-acid sequence alignment of ApHal2 from *A. pullulans* (GenBank Accession No. KC242234), HwHal2A from *H. werneckii* (ABF68774), and Hal2 from *S. cerevisiae* (NP_014577), using ClustalW. The PAP-phosphatase-like domain [cd01517] is shaded in light grey, and includes the conserved active site residues (indicated by the bar), the substrate-binding-site residues (shaded in dark grey), and the putative Li^+^/Na^+^ binding sites (black arrows). The META sequence motif, which is implicated in increased Na^+^ tolerance, is framed. (B) Functional complementation with ApHal2. Ten-fold serially diluted cultures of *S. cerevisiae hal2* mutant cells transformed with the empty plasmid pRD53 or the plasmid carrying *HAL2*, *ApHAL2A* or *HwHAL2* (as indicated) were plated on YNB-Ura (-URA) or YNB-Ura-Met (-URA-MET) plates containing glucose (Glc) or galactose (Gal). The data are representative of three independent experiments.

To identify the complete sequence of *ApHAL2*, which encodes a 3′-phosphoadenosine-5′-phosphatase from *A. pullulans*, we amplified and sequenced the upstream and downstream sequences of the already described partial gene sequence (GenBank: DQ519094) [26], using genome DNA walking. The intron-less gene was named *ApHAL2* (GenBank KC242234), and it encodes a 1071-bp-long coding sequence, which is translated into a 356 amino-acid protein (Fig. 1). The predicted molecular mass of the protein is 37.7 kDa, and the predicted isoelectric point (pI) is 5.3, similar to the pI of 5.2 of HwHal2A (from *H. wernecki*; ABF68774.1), but lower than the pI of 5.8 of Hal2 (from *S. cerevisiae*; NP_014577). ApHal2 shows 73.7% amino-acid identity with HwHal2A and 42.2% amino-acid identity with Hal2 from *S. cerevisiae*. The alignment of ApHal2 with HwHal2A and Hal2 (Fig. 1A) highlighted the PAP-phosphatase-like domain (cd01517), which includes the conserved active-site residues (DPID.TK…H…D…WD), the substrate-binding-site residues (H^242^, K^269^, R^283^, E^292^, D^296^), and the putative Li^+^/Na^+^-binding sites (D^135^, D^296^). Importantly, we also identified the nine-amino-acid sequence (^175^DSEPLTEDL^183^) that is analogous to the META sequence of HwHal2A but not found in Hal2, and which has previously been implicated in increased sodium tolerance of this enzyme, and consequently of the corresponding organism [26].

To determine whether the *ApHAL2* gene is a true functional homologue of the yeast *HAL2* gene, we carried out complementation assays (Fig. 1B). To this end, the *S. cerevisiae hal2* mutant cells were transformed with the pRD53::ApHAL2 plasmid, with pRD53::HAL2 and pRD53::HwHAL2A as the positive controls, or with the pRD53 empty vector as the negative control, with selection on uracil minimal medium. As the only apparent phenotype of *HAL2*-disrupted cells is an auxotrophy for methionine [20], the complementation was assayed on uracil minimal medium plates with no methionine supplementation, and with added Gal to induce gene expression. Expression of *ApHAL2* in the *S. cerevisiae hal2* mutant growing on YNB-Ura-Met Gal plates successfully restored growth to the level observed on control plates YNB-Ura Glc with supplemented methionine. The growth of the strain was slower on YNB-Ura-Met plates with added glucose, which repressed the expression of ApHal2 (Fig. 1B). Similar results were obtained when the *HAL2* or *HwHAL2A* genes were expressed, for which a role in methionine synthesis has already been demonstrated [20], [26]. The *hal2* strain carrying the empty plasmid could not restore growth on any of the plates growth on plates without methionine to the level observed on the control plates (Fig. 1B).

### The role of *ApHAL2* in tolerance to sodium

**Figure 2:**
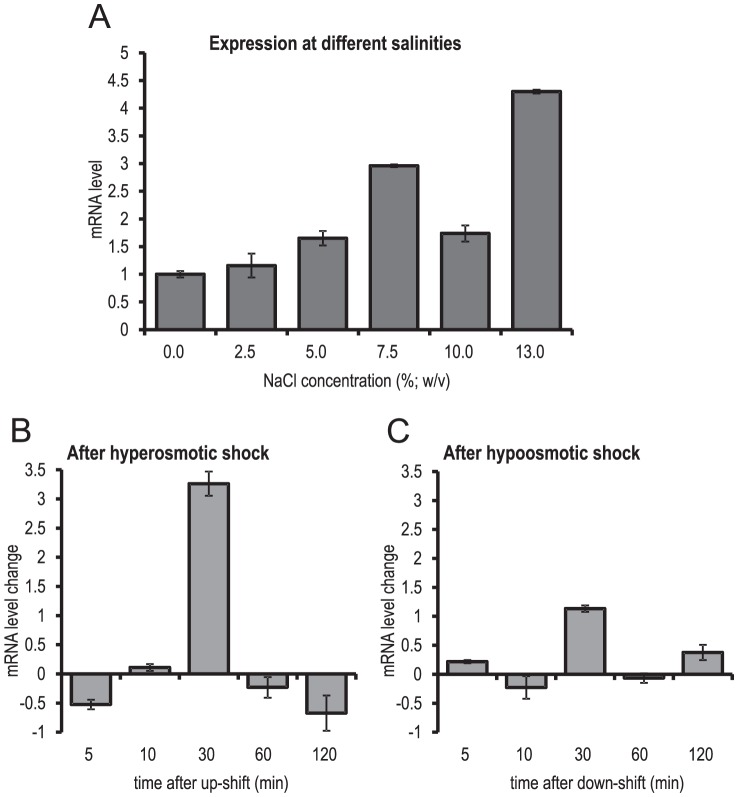
*ApHAL2* gene expression with salt adaptation and under stress conditions. Expression analysis in *A. pullulans* during constant growth at increasing concentrations of NaCl (A), and after hypersaline (B) and hyposaline (C) shock. The data are relative fold-inductions of cDNA levels, as means (±standard deviation) of two qPCR experiments (biological replicates), each carried out in duplicate, relative to no NaCl in adapted cells (A), and relative to zero time in stressed cells (B, C).

The qPCR analysis showed that the expression of *ApHAL2* generally increases with increasing environmental salinity (except at 10% NaCl; w/v). During growth at 13% NaCl (w/v), the expression of *ApHAL2* was more than four-fold higher than in the medium without salt (Fig. 2A). In the 2 h after the *A. pullulans* cells were subjected to hyperosmotic or hypo-osmotic shock by rapidly changing the salt concentration of the medium, no clear changes in expression were observed (Fig. 2B, C). With the exception of increased expression at 30 min after the hyperosmotic shock, the differences in expression were less than two-fold (compared to before the shock). The peak of expression at 30 minutes occurred in both biological replicates, but additional time points would be needed to confirm and investigate this change in more detail.

**Figure 3:**
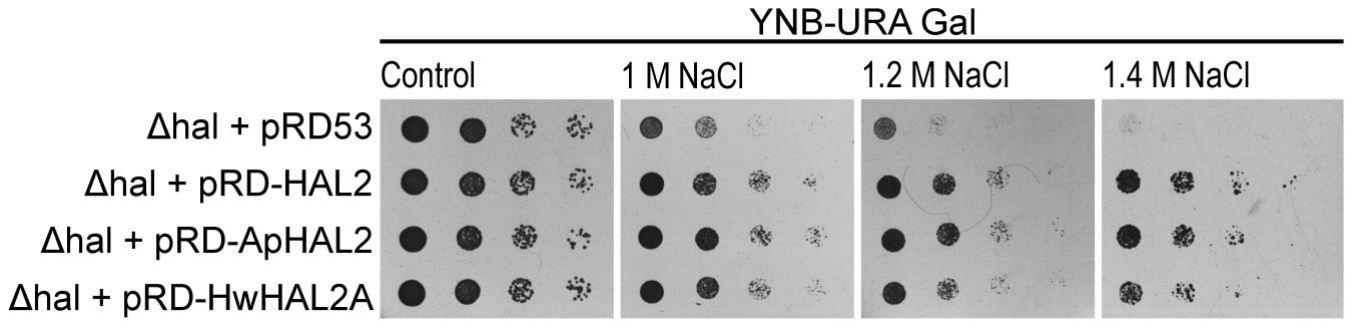
*ApHAL2* salt tolerance plate assay. Ten-fold serially diluted cultures of *S. cerevisiae hal2* mutant cells transformed with the empty plasmid pRD53 or the plasmid carrying *HAL2*, *ApHAL2* or *HwHAL2* (as indicated) were plated on YNB-Ura (-URA) plates containing galactose (Gal), with no NaCl (Control) and with the indicated concentrations of NaCl. The data are representative of three independent experiments.

The role of PAP phosphatases in tolerance to sodium has already been shown for Hal2 from *S. cerevisiae* [20] HwHal2A and HwHal2B from the extremely halotolerant *H. werneckii* [26] and *DHAL2* from halotolerant *D. hansenii* [27]. Therefore we also checked the effects of sodium on the growth of yeast expressing *ApHAL2* (Fig. 3). To this end, we grew the above-described *S. cerevisiae hal2* transformants on uracil minimal medium supplemented with Gal for induction of gene expression (YNB-Ura Gal), and with NaCl added to different concentrations. Mutant strains expressing *ApHAL2* grew at up to 1.4 M NaCl, similar to strains expressing *HAL2* and *HwHAL2A*. The control strain, the *hal2* mutant carrying the empty plasmid, only grew well up to 1 M NaCl (Fig. 3).

### Modified SAL1 *(mSAL1)*

**Figure 4:**
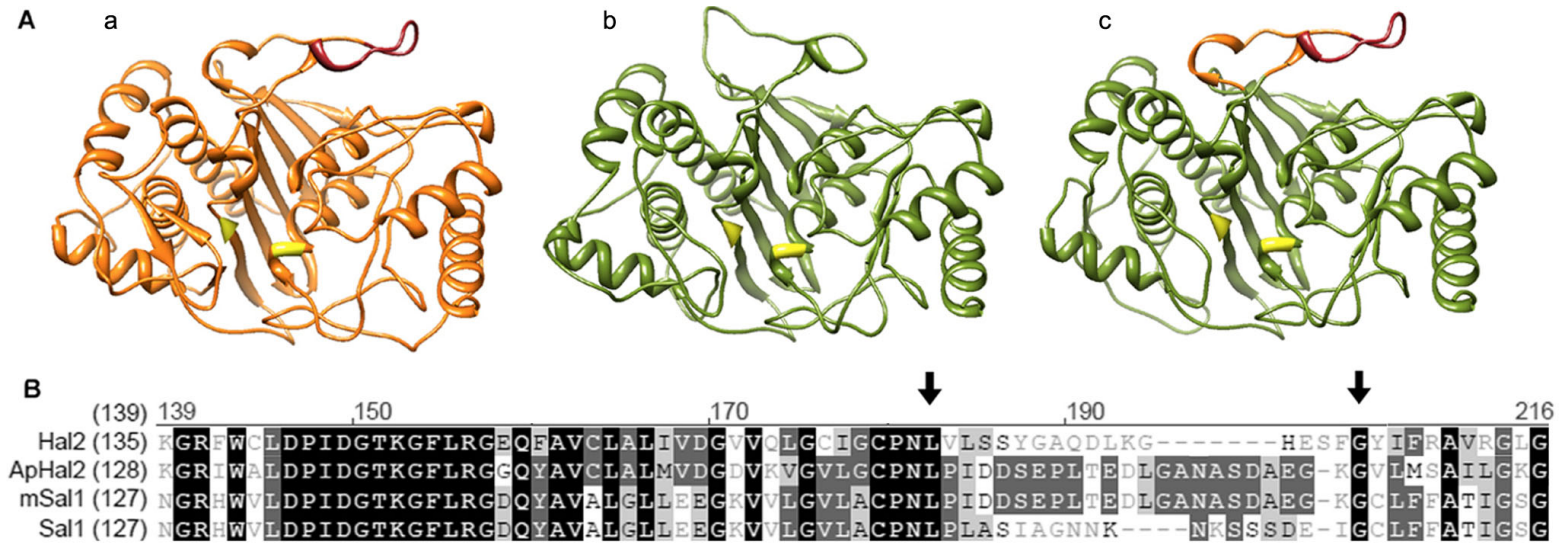
Modelling of a modified SAL1 protein with the inserted loop with the META region from ApHal2. Top: Three-dimensional models of *A. pullulans* ApHal2 (A), *A. thaliana* SAL1 (B), and modified SAL1 (mSAL1) (C) with the replaced loop shown in orange and the META region in red. The conserved active-site amino acids are in yellow. The models were prepared on the basis of the *S. cerevisiae* Hal2 structure, using Swissmodel. Bottom: Amino-acid alignment of Hal2 (NP_014577), ApHal2 (KC242234), mSAL1 and SAL1 (ID 836519), using AlignX. Black arrows indicate the conserved amino acids lysine (170) and glycine (189), bordering on the loop of the SAL1 protein, which was replaced by the loop from ApHal2, containing the META region (DSEPLTEDL).

We have shown that ApHal2 protein is a PAP phosphatase, with the key role in tolerance to environmental sodium. Previous research showed that transfer of META motif from the HwHal2A of the halotolerant *H. werneckii* to Hal2 protein of the salt-sensitive *S. cerevisiae* improves the tolerance of the latter yeast to sodium [26]. Therefore we wanted to check if transfer of the META motif from ApHal2 protein of the halotolerant *A. pullulans* to SAL1 of the salt sensitive *A. thaliana* has a similar effect. To this enda chimeric protein was designed that consisted of *A. thaliana* SAL1 and the META region from *A. pullulans* (Fig. 4). The three-dimensional model of ApHal2 and SAL1 was built in SWISS-MODEL, through homology-based protein structure modelling on the crystallographic template structure of *S. cerevisiae* Hal2 and the ApHal2 and SAL1 sequences, aligned to Hal2 with Align X (Fig. 4A, B, C). Models of Hal2 homologues showed the conserved amino acids of lysine (171) and glycine (189) as an appropriate site for replacing the SAL1 17-amino-acid sequence with the 21 amino acids from ApHal2 (Fig. 4, bottom). This replacement led to a chimeric protein, which we refer to as modified (m)SAL1 (Fig. 4C).

**Figure 5:**
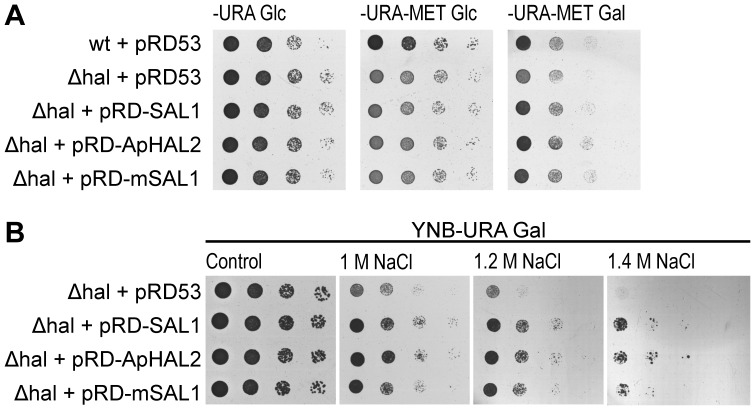
Functional complementation (A) and salt-tolerance plate assay (B) for SAL1 and mSAL1 in *S. cerevisiae hal2* mutant cells. Ten-fold serially diluted cultures of transformed cells with the empty plasmid pRD53 or the plasmid carrying *SAL1*, *ApHAL2* or *mSAL1* (as indicated) were plated on YNB-Ura (-URA) or YNB-Ura-Met (-URA-MET) plates containing glucose (Glc) or galactose (Gal), and no NaCl (Control) or the indicated concentration of NaCl. The data are representative of three independent experiments.

To confirm the functionality of the modified *(m)SAL1* gene, we performed functional complementation (Fig. 5A) and salt tolerance assays (Fig. 5B) in the *S. cerevisiae hal2* mutant cells. To this end, we transformed the mutant strain with the pRD53::mSAL1 plasmid or with pRD53::SAL1 and pRD53:: ApHAL2 as the positive controls, or the pRD53 empty vector as the negative control, and proceeded as described above for ApHal2. Expression of *mSAL1* successfully restored the growth of the mutant on YNB-Ura-Met Gal plates to the level observed on control plates YNB-Ura Glc supplemented with methionine. The growth of the strain was slower on YNB-Ura-Met plates with added glucose, which repressed the expression of *mSAL1*, (Fig. 5A). Similar results were obtained when the *SAL1* or *ApHAL2* genes were expressed, while the *hal2* strain that carried the empty plasmid did not restore growth on plates without methionine to the level observed on the control plates. Modified SAL also conserved the role of SAL1 in tolerance to sodium, as *hal2* mutant strain expressing *mSAL1* could grow on plates with up to 1.4 M NaCl (Fig. 5B), similar to the strains expressing the *SAL1* or *ApHAL2* genes. The control strain, the *hal2* mutant carrying the empty plasmid, only grew well up to 1 M NaCl.

### Arabidopsis plants exposed to salt stress in tissue culture grow better when overproducing *mSAL1*

Plants were transformed for overexpression of *mSAL1* or native *SAL1*, and they showed comparable mRNA expression levels of the inserted genes. In the transformed *SAL1* overexpression lines (sal1 lines), the mRNA levels of *SAL1* were elevated by 10-to-50-fold, and the *mSAL1* mRNA levels in the *mSAL1* overexpression lines (msal1 lines) were 10-to-50-fold higher than for the native *SAL1*. The mRNA expression levels were not dependant on salt stress and did not differ between the leaves, roots and stem tissues (data not shown).

**Figure 6:**
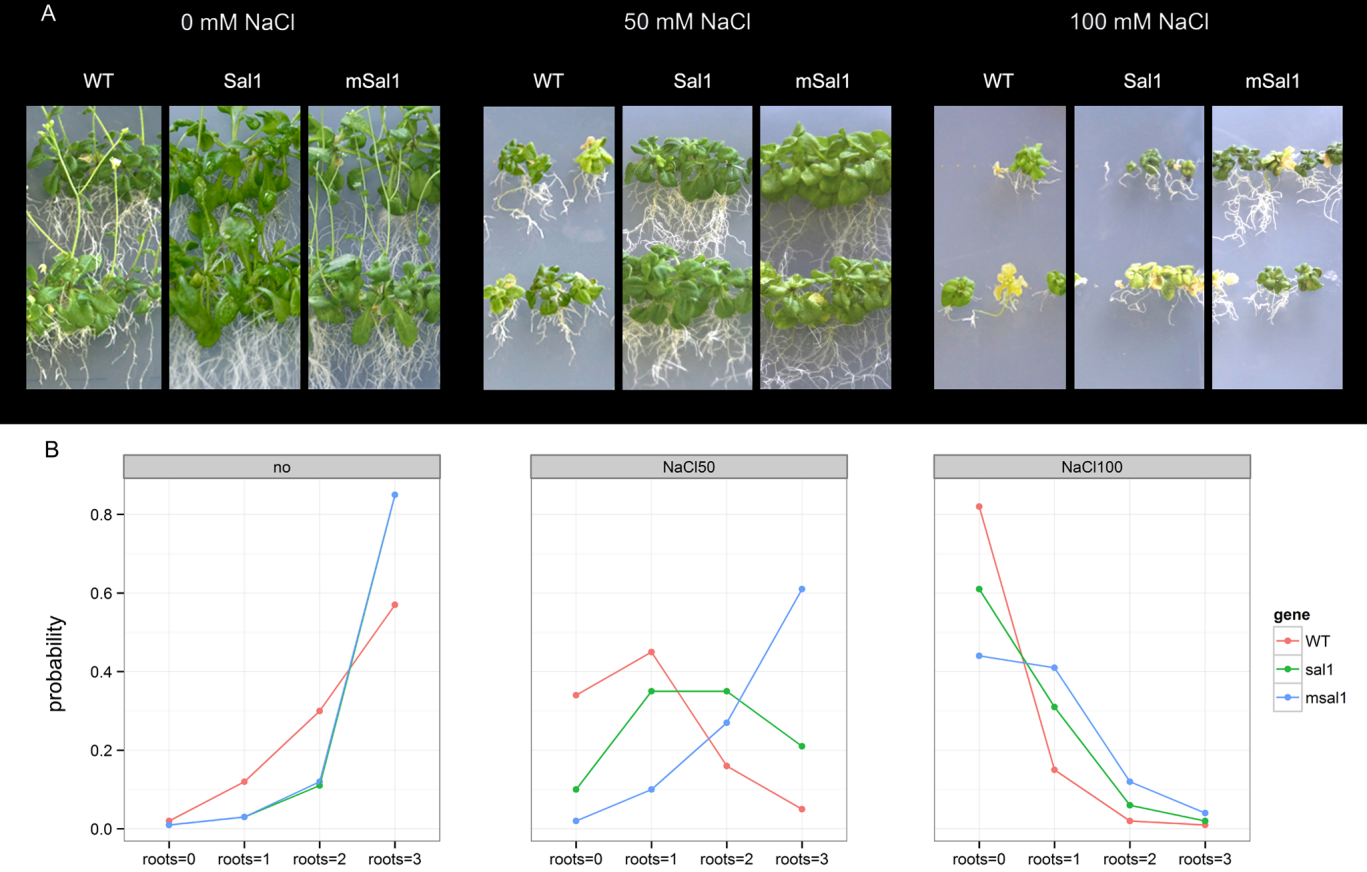
*A. thaliana* plants exposed to salt stress. Wild type plants and plants overexpressing *SAL1* and *mSAL1* (as indicated) were grown for four weeks in medium containing 0, 50 and 100 mM NaCl (as indicated). (A) Representative plants, showing differences in root length under the salt stress. (B) Relative occupancy of root length group – proportion of plants having root length of given category (0, shortest; 3. longest; see Methods). The root lengths were predicted separately from the statistical model for the three genotypes and NaCl concentrations (as indicated), as they were shown to be statistically significant for determining the root length groups (p<0.001).

The msal1 and sal1 lines did not differ in terms of their root growth under normal growth conditions, while the *msal1* lines developed longer roots when grown under salt stress (Fig. 6A). Interestingly, the interactions between the 50 mM NaCl medium and the *msal1* lines were significantly different from *sal1* or the wild-type genotypes (p<0.01). In other words, when growing on a medium with increased NaCl (50 mM), the *msal1* lines were twice as likely to have roots in a higher length category than the *sal1* or wild-type plants (Fig. 6B). In 50 mM NaCl, a root length of ≥2 cm was reached by 88% of the *msal1* lines, and by only 56% of the *sal1* lines. Also, in the presence of 100 mM NaCl, greater numbers of the *msal1* plant lines grew longer roots than for the *sal1* lines or wild-type plants (Fig. 6B, right panel). This was a clear trend which however could not be confirmed as statistically significant since a high rate of dying at 100 mM NaCl did not leave enough living plants which are necessary to compare plant growth levels under varying conditions with the desired confidence level (0.95 or above). A root length of ≥2 cm was reached by 16% of the *msal1* lines and by only 8% of the *sal1* lines and 3% of the wild-type plants.

Similar results were obtained for the leaf surface area as for the root length. On average, the leaf surface area in the *msal1* plant line was significantly greater than in the *sal1* lines (by 3 cm^2^ per plate with 20 plants, which corresponded to a 40% greater total leaf surface area when comparing *msal1* with the *sal1* lines; p<0.01) and in the wild-type (by 4 cm^2^, which corresponds to an increase of 60% in total leaf surface area when comparing *msal1* with the wild-type plants; p<0.01). The *sal1* lines and wild-type plants grew leaves of roughly the same surface areas, and although it did not reach significance, there was a trend towards a greater leaf surface area for the *sal1* lines *versus* the wild-type plants (p = 0.08).

As a consequence of the increased NaCl concentrations, the total leaf surface areas of the plants were reduced. The leaf surface areas in the presence of 50 mM NaCl roughly 80% of the leaf surface in the absence of salt for wild type plants and *sal1* lines and around 85% of the total leaf surface in *msal1* lines (p<0.01). Interestingly, at 100 mM NaCl *sal1* lines and wild type plants again behaved in similar manner having the leaf surface of only 30% of those measured under conditions without salt (p<0.01).

### Drought tolerance

**Figure 7:**
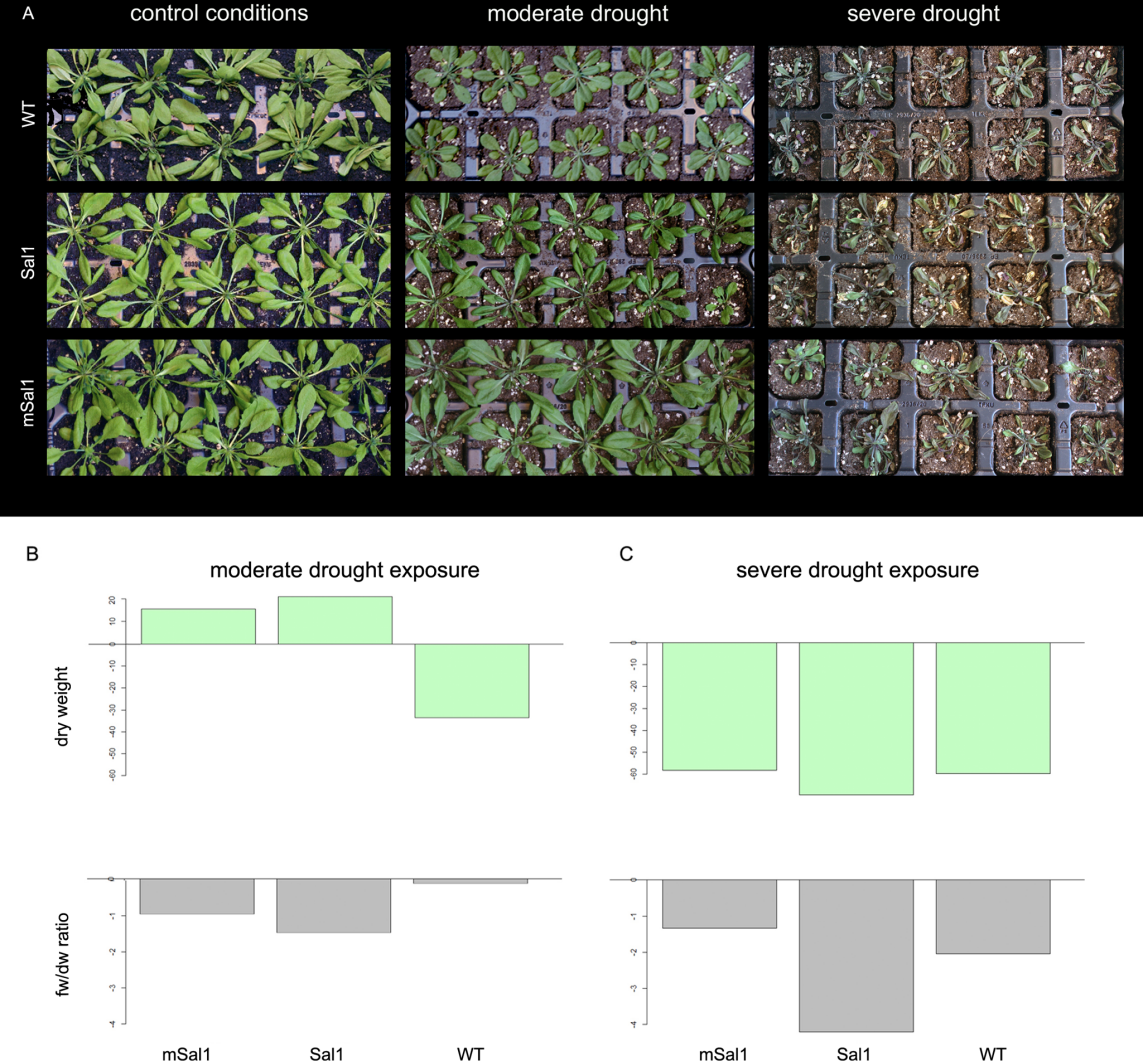
*A. thaliana* plants exposed to drought stress. Plants overexpressing *mSAL1* (msal1) and *SAL1* (sal1) and wild-type plants (WT) were grown under control conditions (6 weeks old), and under moderate (6 weeks old) and severe (7 weeks old) drought (as indicated). (A) Representative plants, watered normally (control) or under moderate drought (two weeks without watering) or severe drought (three weeks without watering). (B) Differences in plant weight; the weights of revitalized plants (i.e. after moderate/severe drought) were deduced from weight of plants which were grown in normal conditions. Positive difference values indicate a successful revitalization. Dry weight is shown on the top plots and fw/dw ratio is plotted in the bottom for moderate and severe drought, respectively.

When exposed to moderate drought and subsequent revitalisation, only the wild-type plants showed a decreased dry weight, while both the *msal1* and *sal1* lines showed increased end dry weight in comparison with plants that were watered normally (Figs. 7A and 7B upper left panel). This increase in dry weight was around 16 mg for both of these genotypes, which represented a 14% increase in the *msal1* lines and an 18% increase in the *sal1* lines (p = 0.0001). In other words, although, in general, the dry weights of the plants did not differ between genotypes while being watered normally (p = 0.16), it was the *msal1* and *sal1* lines that showed increased dry weight after moderate drought exposure (p = 0.0001 and positive difference between weights, Fig. 7B upper left panel). Regardless of the tested genotype, the fresh weight to dry weight (fw/dw) ratios were significantly lower after moderate drought exposure (p = 0.0001), with all three of the genotypes responding in the same manner, i.e. by lowering their respective fw/dw ratio (Fig. 7B, lower left panel).

After exposure to severe drought and the subsequent revitalisation, the dry weights were significantly lower in all of the genotypes, by around 40% (p<0.0001). Indeed, interesting behaviour was observed when the plants were exposed to this severe drought. Although the fw/dw ratio was not different between the genotypes, the revitalisation ability of the *sal1* lines was lower compared to the *msal1* lines and the wild-type plants (see Fig. 7B, lower right panel). For 15% of the *sal1* lines, the long drought exposure was nearly fatal, and despite subsequent revitalisation, they did not rehydrate like the other plants. It appears that *sal1* lines cannot tolerate long drought exposure, as their fw/dw ratio showed a higher rate of decrease (p<0.0001) than the *msal1* and wild-type lines, which behaved similarly (Fig. 7B, lower right panel).

## Discussion

Genetic modifications have been extensively used to improve the quality and yield of crops, including increased plant tolerance to saline stress. One approach is based on the identification of the crucial metabolic reactions and the corresponding enzymes that are specifically sensitive to inhibition by Na^+^ [22], and their complementation with ion-tolerant homologues from other species. For the yeast *S. cerevisiae*, the enzyme determining the Na^+^ toxicity of the cells is the PAP phosphatase Hal2 [22], homologues of which have been identified in many fungi [26]–[28] and plants [7], and which are highly conserved. In the present study, we characterised ApHal2, a Hal2 homologue, in a polyextremotolerant ascomycetous fungus *A. pullulans*. This black-yeast-like organism shows great adaptability and can grow under a wide variety of stress conditions, including at 17% NaCl (w/v) (reviewed in [42]), which makes this species a promising gene donor for improvement of the stress tolerance of plants. We have shown that ApHal2 can complement the role of Hal2 in methionine biosynthesis and recovers the growth of the *Δhal2 S. cerevisiae* mutant in media without methionine. Its gene expression in *A. pullulans* is salt dependent. Similar to HwHal2A, ApHal2 rescues the tolerance of the *Δhal2* yeast mutant to sodium. Based on the previous data on HwHal2A, this is likely to be due to the nine-amino-acid stretch known as the META motif. When the META motif was inserted into the *A. thaliana SAL1*, a homologue of ApHal2, and when this modified *SAL1* was overexpressed in transgenic plants, the plants showed improved halotolerance and moderate drought tolerance. When exposed to salt stress, these plants grew longer roots and had greater leaf surface areas in comparison with the plants overexpressing the native *SAL1* and the wild-type *Arabidopsis*. Similarly, after moderate drought exposure, the plants expressing the native and modified *SAL1* increased in dry weight by more than the wild-type plants.

ApHal2 is highly similar to the previously described HwHal2 proteins from the extremely halotolerant *H. werneckii* [26], and it also contains the nine-amino-acid META motif, which was first identified in the PAP-phosphatase-like domain of the HwHal2 proteins [26]. This region was later shown to be present in the majority of the other Hal2 proteins from halotolerant melanised fungi, like *A. pullulans*, *Phaeotheca triangularis*, *Trimmatostroma salinum* and *Cladosporium sphaerospermum*, but was absent from the salt sensitive *S. cerevisiae* and the halotolerant saccharomycete *D. hansenii* [26]. Deletion of the META region from *HwHAL2B* decreased the beneficial effect of this protein on the halotolerance of *S. cerevisiae*, whereas insertion of the META region into the native *S. cerevisiae* Hal2 improved the halotolerance, which indicates the importance of this region for the HwHal2B enzyme tolerance to Na^+^ [26]. When comparing ApHal2 to the SAL1 protein, which is a homologue in *A. thaliana*, the active-site residues, substrate-binding-site residues, and putative Li^+^/Na^+^ binding sites are all conserved. The variability is the highest in the region where the META sequence is inserted in ApHal2 but absent in SAL1. SAL1 is one of five *Arabidopsis* homologues of Hal2, with the others being SAL2, SAL3, SAL4 and AtAHL [7], [17], [18]. *SAL1* and *AtAHL* are transcribed at much higher levels than the other family members (www.genevestigator.com), although SAL1 has been shown to be the major enzyme that hydrolyses PAP *in planta* [19], and it also has a much higher affinity and activity toward PAP than AtAHL [17].

It has already been proposed that expression of the Hal2 protein or its homologues might improve plant resistance towards salt or drought stress [7], [10], [43], and a tomato expressing the yeast *HAL2* gene showed increased halotolerance [25]. Therefore, we tested the beneficial effects of expression of the halotolerant *ApHAL2* homologue in plants. Due to assumptions that the SAL1 protein is involved in plant stress signalling [11], [13], we decided to maintain the major part of the native SAL1 protein intact, and thus to minimise the interference with possible interactions with other proteins. Instead of expressing the whole *ApHAL2* gene, we constructed a chimeric mSAL1 protein, which contained the 21-amino-acid stretch including the META motif from the ApHal2 protein. Similarly, in a previous study the META sequence from HwHal2 was inserted into ScHal2, and this significantly increased the salt tolerance of *S. cerevisiae* [26]. The functionality of the chimeric mSAL1 was confirmed, as the protein complemented the role of Hal2 in methionine biosynthesis and rescued the salt tolerance of the *Δhal2* mutant. Similarly, we and others have shown the functionality of native SAL1 [7]. It has also been shown that, *vice versa*, Hal2 can partly complement *sal1* plant lines [12], although the drought response was not measured.

As has been proposed for drought stress responses, PAP accumulates and moves from the chloroplasts to the nucleus, where it activates stress-responsive genes, while SAL1 is a negative regulator of PAP that is localised in the chloroplasts and mitochondria [13]. It is believed that it is the absence of SAL1 that increases the drought tolerance in plants [13], [44], [45], which is based on experiments that have used *Arabidopsis* mutants that lack any detectable SAL1. However, the plant response was only measured under severe drought conditions, and might not be relevant for moderate drought responses, as it has been observed that the physiological responses to moderate and severe drought differ significantly [46]. Thus, by exposing plants to both moderate and severe drought, we have demonstrated that the elevated SAL1 levels or the newly expressed *mSAL1* are actually beneficial under moderate drought conditions. The findings of a severe drought effect on *SAL1*-expressing plants are consistent with previous studies [13], [44], [45]. However, plants that express *mSAL1* with the META region from ApHal2 did not respond in the same manner. While moderate drought and subsequent revitalisation affected the wild-type plants by causing a significant drop in their dry weight, the situation was reversed for the transgenic plants overexpressing the native or the modified *SAL1*. Surprisingly, both the *mSAL1* and *SAL1* overexpression lines (sal1 and msal1 lines) showed a significant increase in dry weight after exposure to moderate drought conditions (14% and 18%, respectively), with the *msal1* lines showing the highest end dry weight. This confirms that both *mSAL1* and *SAL1* overexpression contribute to the increased tolerance to moderate drought, and hence might be beneficial for plant breeding. Under severe drought stress, the *sal1* lines were more affected by drought than the *msal1* or wild-type lines. Approximately 15% of the *sal1* lines failed to rehydrate, and they also had lower dry weights and lower fw/dw ratios.

Although Hal2 and SAL1 both hydrolyse PAP, their metabolic roles might be different. In yeast, PAP is a by-product in sulphur assimilation, and deletion of Hal2 results in the accumulation of PAP, which will in turn inhibit sulphur assimilation, and result in methionine auxotrophic growth [20], [47]. Higher plants, however, do not use PAP and 3′-phosphoadenosine 5′-phosphosulphate in sulphur assimilation, but reduce sulphate through adenosine-5′-phosphosulphate reductase [48], [49]. Therefore, it has been proposed that PAP might not interfere with sulphur assimilation in *Arabidopsis* [19], but will have a key role in stress signalling [13]. Although it was previously thought that fungal PAP phosphatases can only have PAP phosphatase activity [26]–[28], it was later shown that the enzyme Tol1 also has an inositol 1,4-bisphosphate phosphatase activity [28]. This might be true for ApHal2, as it is more similar to fission yeast Tol1 than to the Hal2 enzyme (as % identity). In this case, ApHal2 might act on inositol 1,4-bisphosphate and inositol 1,3,4-trisphosphate, and plants that express *mSAL1* might compensate for the delay in the drought response by the initiation of the signalling cascade via inositol 1,4,5-trisphosphate.

Unlike drought stress, salt stress in plants does not result in increased PAP levels [16], and *Arabidopsis* seedlings that lack SAL1 show only moderate sensitivity to salt stress [50]. Nevertheless, just as for the yeast Hal2 [21], plant SAL1 is sensitive to increased NaCl concentrations *in vitro* (IC_50_ = 200 mM), albeit much less so than another *Arabidopsis* homologue, AtAHL [7], [17]. *Arabidopsis* mutants that lack SAL1 and grow in tissue culture have been reported to be more sensitive to salt stress [10], and to have reduced root development [14] and changed leaf morphogenesis [15]. We have shown here that overexpression of the native *SAL1* does not enhance the salt tolerance of the transgenic plants, which was also shown in another study [19]. *SAL1* overexpression had no influence on plant root formation, while the total leaf surface area of the *sal1* lines was slightly greater than that of the wild-type plants, although the difference was not statistically significant. However, plants expressing *SAL1* with the META sequence showed a statistically significant increase in salt tolerance. Under normal growth conditions, the *msal1* and *sal1* plant lines did not differ in terms of their root growth, while under salt stress, the *msal1* lines developed longer roots, both on 50 mM and 100 mM NaCl. Similar results were obtained for the leaf surface area: regardless of the growth conditions, the leaf surface area in plant *msal1* lines was significantly greater than in *sal1* lines and wild-type plants.

It is still unknown how the presence of META motif influences the enzyme tolerance to Na^+^. Its effect might result from changes in the size and/or geometry of the Mg^2+^-binding site 2, where Na^+^ or Li^+^ can also bind. The analysis of random mutations of Hal2 identified a salt bridge, between Glu238 and Arg152, and a hydrophobic bond, between Val70 and Trp293, as important framework interactions in the determination of cation sensitivity [51]. Hal2 proteins mutated in these sites and the native mammalian enzymes where the salt bridge is not present are Na^+^ resistant [51]. Similarly, mutating the residues that form the salt bridge in DhHal2, a Hal2 homologue from *D. hansenii*, increased the tolerance to Li^+^ by 14-fold and to Na^+^ by 3-fold [52]. A similar mechanism might cause increased resistance of the mSAL1 protein to Na^+^ upon the insertion of the META motif in *A. thaliana*. The functions of such mSAL1 would not be affected by Na^+^ and consequently the exonucleases, which in wild-type plants are inhibited under high-salt conditions by PAP, would stay functional. Similarly, all of the downstream targets of these enzymes, such as micro (mi)RNA and small-interfering (si)RNA turnover [8] and activation of the ENA ion transporters [7], would also function correctly at increased salt levels.

We have shown that not only proteins from halotolerant fungi, but even parts of these proteins can have beneficial effects on plants exposed to high salinity. This is an important discovery, as genetic resources for improving salt and drought tolerance of crops are much needed [3], [53]. The obstacles that previously hindered the use of naturally stress-tolerant fungi for this purpose are being quickly overcome by the expanding knowledge of fungal stress tolerance, by the advances in the methodologies of large-scale screening for useful traits, and by the growing number of genome and transcriptome sequencing projects of the species in question [54]. Determinants of stress tolerance from these fungi, such as the here-described 3′-phosphoadenosine-5′-phosphatase, can alleviate some of the current major agricultural problems, and should be considered as a resource for improving stress-tolerance in plant breeding in the future.

## Supporting Information

Table S1 Primers used in the cloning, and primers and probes used in the qPCR assays, with the corresponding coefficients of determination (R^2^) and the slopes of the standard curves (k) used for the reaction efficiency calculations. The ApHAL2 primers contain EcoRI and BamHI restriction sites (underlined). *SAL1* and *mSAL1* primers contain SmaI and KpnI restriction sites, respectively (underlined). All primers and probes were developed within this study, except for Cox _P, which was designed previousely [55].
(DOCX)

Table S2 Amino-acid sequence of the chimeric mSAL1 protein and the corresponding nucleotide sequence. The chimeric protein consists of the SAL1 protein from *Arabidopsis* and a 21-amino-acid stretch from yeast *A. pullulans* (in bold) that includes the META region (underlined). In the nucleotide sequence codon, the use was optimised for expression in *Arabidopsis* (in bold).
(DOCX)
