# Supplementary material for: Insertion of a Specific Fungal 3′-phosphoadenosine-5′-phosphatase Motif into a Plant Homologue Improves Halotolerance and Drought Tolerance of Plants
Source: PLoS One. 2013 Dec 9;8(12):e81872. doi: 10.1371/journal.pone.0081872 (PMC3857206; doi:10.1371/journal.pone.0081872)
Supplement: Table S1 — Primers used in the cloning, and primers and probes used in the qPCR assays, with the corresponding coefficients of determination (R2) and the slopes of the standard curves (k) used for the reaction efficiency calculations. The ApHAL2 primers contain EcoRI and BamHI restriction sites (underlined). SAL1 and mSAL1 primers contain SmaI and KpnI restriction sites, respectively (underlined). All primers and probes were developed within this study, except for Cox _P, which was designed previousely [55]. (DOCX) [file pone.0081872.s001.docx]

**Table S1.** Primers used in the cloning, and primers and probes used in the qPCR assays, with the corresponding coefficients of determination (R^2^) and the slopes of the standard curves (k) used for the reaction efficiency calculations. The ApHAL2 primers contain EcoRI and BamHI restriction sites (underlined). *SAL1* and *mSAL1* primers contain SmaI and KpnI restriction sites, respectively (underlined). All primers and probes were developed within this study, except for Cox _P, which was designed previousely [55].

| **Assay** | **Primer/ probe** | **Sequence (5'-3')** | **k** | **R^2^** |
| --- | --- | --- | --- | --- |
|  | **GenomeWalker** |  |  |  |
| gwaph2U | gwaph2U | AGTCAGAGGCTCGCTGTCGTCG |  |  |
|  | gwaph2Un | CTTGACGTCGCCGTCGACCATC |  |  |
| gwaph2D | gwaph2D | GACTCGCAAGCCAAGTACGCATCC |  |  |
|  | gwaph2Dn | CGTCTTCCCGTCAAGAAGGACTACC |  |  |
|  | **PCR** |  |  |  |
| aphal2 | aphal2_F | TCAGGAATTCATGTCGACCGACTACTCGAA |  |  |
|  | aphal2_R | CATAGGATCCTTACTGCTTGGCCGAGAGAA |  |  |
| sal1_sc | sal1_sc_F | ACCCGGGATGGCTTACGAGAAAGAGCTT |  |  |
|  | sal1_sc_R | AGGTACCCTAAAGAGCTGAAGCTTTCT |  |  |
| hal2 | hal2_F | ACCCGGGATGGCATTGGAAAGAGAA |  |  |
|  | hal2_R | AGGTACCTTAGGCGTTTCTTGACTGAAT |  |  |
| sal1 | Sal1_F | CACCATGGCTTACGAGAAAGAGC |  |  |
|  | Sal1_R | TAAGAGCTGAAGCTTTCTCTTGCTC |  |  |
|  | **qPCR** |  |  |  |
| aphal2 | aphal2_F | GTGTCCTCATGTCCGCCATCCT | -3.4 | 0.998 |
|  | aphal2_R | ATGGAGATGGTGGTGGCGTTCT |  |  |
| sal1 | (m)Sal1_P | [FAM]-TGTGCTTGCTTGTCCAAACTTGCCG-[TAMRA] | -3.4 | 0.993 |
|  | (m)Sal1_F | GCTCGAGGAAGGGAAAGTAGTTT |  |  |
|  | Sal1_R | TCTGACGAAGATTTGTTCTTGTTGT |  |  |
| msal1 | (m)Sal1_P | [FAM]-TGTGCTTGCTTGTCCAAACTTGCCG-[TAMRA] | -3.4 | 0.998 |
|  | (m)Sal1_F | GCTCGAGGAAGGGAAAGTAGTTT |  |  |
|  | mSal1_R | TCAGTAAGTGGCTCAGAATCATCAA |  |  |
| cox | Cox _P | [FAM]-TGCTTACGCTGGATGGAATGCCCT-[TAMRA] | -3.5 | 0.988 |
|  | Cox _F | TCAGGTATGCCACGTCGTATTC |  |  |
|  | Cox _R | CGGATATATAAGAGCCAAAACTGGAA |  |  |
