# Supplementary material for: Insertion of a Specific Fungal 3′-phosphoadenosine-5′-phosphatase Motif into a Plant Homologue Improves Halotolerance and Drought Tolerance of Plants
Source: PLoS One. 2013 Dec 9;8(12):e81872. doi: 10.1371/journal.pone.0081872 (PMC3857206; doi:10.1371/journal.pone.0081872)
Supplement: Table S2 — Amino-acid sequence of the chimeric mSAL1 protein and the corresponding nucleotide sequence. The chimeric protein consists of the SAL1 protein from Arabidopsis and a 21-amino-acid stretch from yeast A. pullulans (in bold) that includes the META region (underlined). In the nucleotide sequence codon, the use was optimised for expression in Arabidopsis (in bold). (DOCX) [file pone.0081872.s002.docx]

**Table S2.** Amino-acid sequence of the chimeric mSAL1 protein and the corresponding nucleotide sequence. The chimeric protein consists of the SAL1 protein from *Arabidopsis* and a 21-amino-acid stretch from yeast *A. pullulans* (in bold) that includes the META region (underlined). In the nucleotide sequence codon, the use was optimised for expression in *Arabidopsis* (in bold).

| **Name** | **Sequence** |
| --- | --- |
| mSal1 | MAYEKELDAAKKAASLAARLCQKVQKALLQSDVQSKSDKSPVTVADYGSQAVVSLVLEKELSSEPFSLVAEEDSGDLRKDGSQDTLERITKLVNDTLATEESFNGSTLSTDDLLRAIDCGTSEGGPNGRHWVLDPIDGTKGFLRGDQYAVALGLLEEGKVVLGVLACPNLP**IDDSEPLTEDLGANASDAEGK**GCLFFATIGSGTYMQLLDSKSSPVKVQVSSVENPEEASFFESFEGAHSLHDLSSSIANKLGVKAPPVRIDSQAKYGALSRGDGAIYLRFPHKGYREKIWDHVAGAIVVTEAGGIVTDAAGKPLDFSKGKYLDLDTGIIVANEKLMPLLLKAVRDSIAEQEKASAL |
| *mSal1* | Atggcttacgagaaagagcttgatgctgctaagaaagctgcttcactcgctgctcgtctctgtcagaaagttcaaaaggctttgttgcaatcagatgtgcaatcaaaatctgataaaagtccagtgaccgttgctgattatggttcacaagcagttgttagtttagtcttagaaaaagagctcagttctgaacccttttcattggtggctgaagaggactcaggcgatctacgcaaggatggttctcaggatactctggagcgcatcacaaaactcgtgaacgacactttggctaccgaggaatcgtttaatggctctactttgtctactgatgatctacttagagccattgactgtggaacatctgaaggtggtccaaatggtcgacactgggtcttggatccaattgatggcactaaaggatttctgaggggagatcaatacgcagtagcactaggattgctcgaggaagggaaagtagttttaggtgtgcttgcttgtccaaacttgccg**attgatgattctgagccacttactgaggatcttggagctaacgcttctgatgctgagggaaag**ggatgcctcttctttgctacaattggttcagggacatatatgcagctcctagattcaaaatcttctcctgtaaaagtgcaagtctctagtgttgagaatcctgaagaggcatcgttcttcgagtcattcgaaggagctcactctctacatgacttatccagctccattgccaataaactcggtgtcaaagctccaccagtccgtattgatagccaagcaaagtatggagctttatcaagaggagatggagctatatacttacggtttcctcataaaggataccgcgaaaagatttgggaccatgtcgctggtgctatagttgttacagaggcgggtggaatagtgacagatgcagcaggaaagccactggatttctcgaaagggaagtatcttgatttggacacaggcattatcgttgctaacgagaagctaatgcctctgcttttgaaagcagttcgtgactccatagctgagcaagagaaagcttcagctctt |
